# Microscopic Observation Drug Susceptibility Assay for Rapid Diagnosis of Lymph Node Tuberculosis and Detection of Drug Resistance

**DOI:** 10.1128/JCM.02227-15

**Published:** 2015-12-30

**Authors:** Daniela E. Kirwan, Cesar Ugarte-Gil, Robert H. Gilman, Luz Caviedes, Hasan Rizvi, Eduardo Ticona, Gonzalo Chavez, José Luis Cabrera, Eduardo D. Matos, Carlton A. Evans, David A. J. Moore, Jon S. Friedland

**Affiliations:** a Department of Medical Microbiology, St. George's Hospital, London, United Kingdom; b Infectious Diseases and Immunity, Imperial College London, London, United Kingdom; c Instituto de Medicina Tropical Alexander von Humboldt, Universidad Peruana Cayetano Heredia, Lima, Peru; d Department of International Health, Johns Hopkins University, Baltimore, Maryland, USA; e Laboratorio de Investigación en Enfermedades Infecciosas, Universidad Peruana Cayetano Heredia, Lima, Peru; f Department of Cellular Pathology, Barts Health NHS Trust, London, United Kingdom; g Infectious Diseases and Tropical Medicine Unit, Hospital Nacional Dos de Mayo, Lima, Peru; h Universidad Nacional Mayor de San Marcos, Lima, Peru; i Universidad de San Martin de Porres, Lima, Peru; j Department of Pulmonology, Hospital Daniel Alcides Carrión, Callao, Peru; k Infectious Diseases Unit, Hospital Nacional Arzobispo Loayza, Lima, Peru; l Infectious Diseases and Immunity, and Wellcome Trust Centre for Global Health Research, Imperial College London, London, United Kingdom; m IFHAD: Innovation for Health and Development, Universidad Peruana Cayetano Heredia, Lima, Peru; n TB Centre, London School of Hygiene & Tropical Medicine, London, United Kingdom

## Abstract

In this study, 132 patients with lymphadenopathy were investigated. Fifty-two (39.4%) were diagnosed with tuberculosis (TB). The microscopic observation drug susceptibility (MODS) assay provided rapid (13 days), accurate diagnosis (sensitivity, 65.4%) and reliable drug susceptibility testing (DST). Despite its lower sensitivity than that of other methods, its faster results and simultaneous DST are advantageous in resource-poor settings, supporting the incorporation of MODS into diagnostic algorithms for extrapulmonary TB.

## TEXT

In 2013, 14.5% of new tuberculosis (TB) notifications worldwide were extrapulmonary (1), and in certain regions this percentage was much higher (2). Nonspecific disease manifestations and paucibacillary infection make diagnosing extrapulmonary TB challenging (3, 4). Culture, the diagnostic gold standard, allows identification to the species level and drug susceptibility testing (DST) (5), but generating results takes several weeks; automated liquid culture systems are relatively faster (6), but financial constraints limit their use.

The microscopic observation drug susceptibility (MODS) assay is a low-cost, liquid culture-based diagnostic assay for TB (7, 8). With accuracy comparable to that of other culture techniques (7, 9, 10), MODS is faster (7), provides simultaneous DST (10–13), and has World Health Organization (WHO) approval for direct testing of sputum specimens in low-resource settings (14, 15). MODS accurately diagnoses TB from cerebrospinal fluid (16) and pleural specimens (17), but its role in the diagnosis of solid tissue TB remains unknown. This prospective cross-sectional study was designed to investigate the use of MODS for culture of lymph node tissue in an operational setting.

Patients ≥18 years old with lymphadenopathy requiring diagnostic tissue sampling were recruited consecutively from three public hospitals in Lima, Peru, over 14 months. Ethical approval was obtained from the Institutional Ethics Committee of the Universidad Peruana Cayetano Heredia, Asociación Benéfica PRISMA, and each hospital's ethics approval committee. All patients provided written informed consent. For each patient, clinical and demographic data were collected and the treating physician was asked to give the most likely diagnosis. Patients with unknown HIV status were offered testing.

**FIG 1:**
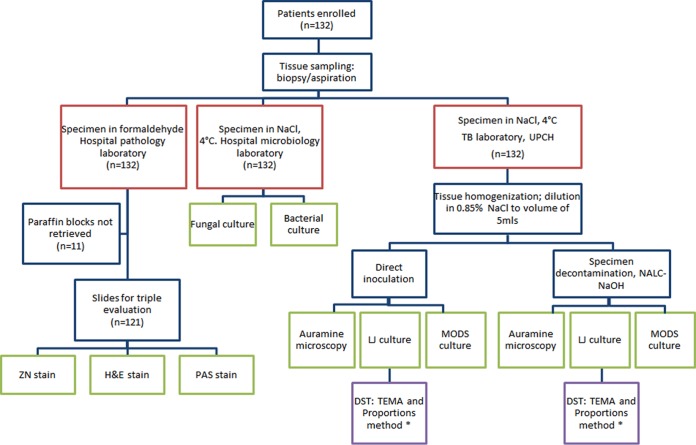
Flow diagram indicating procedures following patient enrollment. For auramine microscopy, the visualization of one or more AFB per 100 fields was considered positive (27). DST of all samples positive by LJ culture was performed. One strain per patient was tested. UPCH, Universidad Peruana Cayetano Heredia; NALC, *N*-acetyl-l-cysteine; H&E, hematoxylin and eosin; PAS, periodic acid-Schiff.

Tissue sampling was performed routinely, and samples were immediately divided into three equal parts and processed as outlined in Fig. 1. MODS was performed in accordance with published standard operating procedures (18). The microbiological criterion for TB was positivity by at least one of the following: auramine microscopy, MODS, or Löwenstein-Jensen (LJ) culture. Strains obtained by LJ culture underwent phenotypic DST by the proportion method, which was performed at the national TB reference laboratory, and the in-house tetrazolium microplate assay (TEMA). Samples for histological evaluation were sealed in paraffin blocks and reported routinely. Once recruitment was ended, the blocks were retrieved and slides were fixed and stained with hematoxylin-eosin, Ziehl-Neelsen (ZN), and periodic acid-Schiff stains. Three independent pathologists blind to the clinical data recorded the presence of acid-fast bacilli (AFB), granulomas, and caseating necrosis and gave an overall diagnosis. A histological definition of TB required concordance between two or more pathologists; when retrieval of paraffin blocks was not possible (*n* = 11), the hospital pathology report was obtained, and if TB was identified, this was used. TB was diagnosed when microbiological and/or histological criteria were met.

Data were entered into Excel and analyzed by Stata version 12 (StataCorp). Nominal demographic data and test characteristics were compared by Fisher's exact test. Times to results were compared by the Mann-Whitney U test. A *P* value of <0.05 was considered significant. Agreement between pathologists was assessed by using Cohen's kappa coefficient for multiple ratings; a kappa value of ≥0.81 was taken to indicate substantial agreement (19).

**FIG 2:**
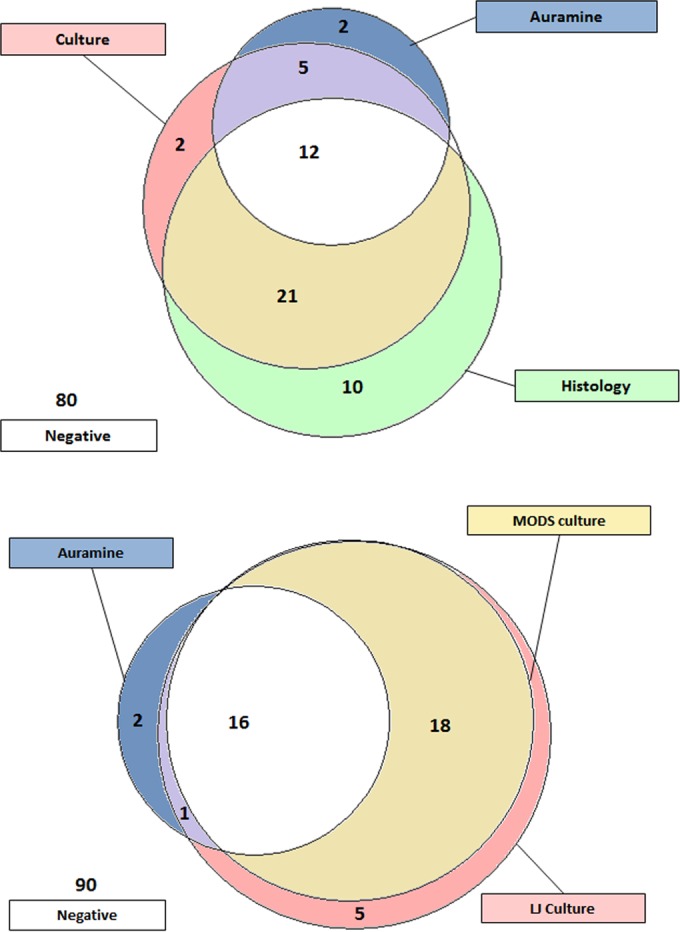
Venn diagrams showing numbers of patients positive by different test modalities. (Top) Positivity for TB by auramine microscopy, TB culture, and histological evaluation (*n* = 132). Twelve patients were positive by all three methods, 26 were positive by two methods, and 14 were positive by only one method. Two patients were positive only by auramine microscopy. (Bottom) Positivity by microbiological methods of TB detection (*n* = 132). All patients positive by MODS were also positive by LJ culture. Eight patients were positive by auramine microscopy (*n* = 3) and/or LJ culture (*n* = 6) but not by MODS.

One hundred forty-four specimens from 132 patients were tested. Patient demographics are presented in Table 1. Fifty-two patients (39.4%) were diagnosed with TB (Table 1; Fig. 2, top). Nineteen were positive by auramine microscopy, 34 were positive by MODS, 40 were positive by LJ culture, and 43 were positive by histology; the sensitivities were 36.5, 65.4, 76.9, and 82.7%, and the negative predictive values (NPVs) were 70.8, 81.6, 87.0, and 89.9%, respectively. HIV-positive patients were more likely than HIV-negative patients to have positive auramine microscopy results (65 and 18.8%, respectively; *P* = 0.001). TB was detected in 5 of 12 patients already undergoing TB therapy (5 by microbiological methods and 3 by histological analysis; all 5 were HIV positive with CD4^+^ counts of <250/mm^3^). Physicians suspected TB in 48 TB-positive and 53 TB-negative patients; thus, the sensitivity, specificity, positive predictive value (PPV), and NPV of the physician's presumptive TB diagnosis were 92.3, 33.8, 47.5, and 87.1%, respectively.

**TABLE 1:** Demographic information and test results for all study participants

Parameter	TB positive (*n* = 52)	TB negative (*n* = 80)	*P* value	Auramine positive (*n* = 19)	MODS positive (*n* = 34)	LJ positive (*n* = 40)	Histological diagnosis of TB (*n* = 43)	Total (*n* = 132)
No. (%) of females^*a*^	23 (44.2)	34 (42.5)	0.86	3 (15.8)	13 (38.2)	16 (40.0)	22 (51.2)	57 (43.2)
Median age, yr (IQR)^*b*^	39 (26–46)	41 (27–55)	0.38	32 (29–40)	32 (25–48)	32.5 (25–46)	35 (24–47)	40 (25–52)
No. (%) HIV positive^*a*^	20 (38.5)	34 (42.5)	0.72	13 (68.4)	12 (35.3)	14 (35.0)	15 (34.9)	54 (40.9)
Median no. of CD4^+^ cells/mm^3^ (IQR)^*b*^	75 (27–218)	163 (121–271)	0.18	87 (25–140)	75 (22–163)	87 (25–140)	63 (25–219)	156 (41–234)
No. (%) with positive sputum smear^*a*^	4 (7.7)	0 (0)	0.02	4 (2.1)	3 (8.8)	4 (10.0)	3 (7.0)	4 (3.0)
No. (%) on TB treatment for >1 wk^*a*^	5 (9.6)	7 (8.8)	1.00	5 (26.3)	4 (11.8)	4 (10.0)	3 (7.0)	12 (9.7)
No. (%) with previous TB^*a*^	10 (19.2)	13 (16.3)	0.65	3 (15.8)	2 (5.9)	4 (10.0)	7 (16.3)	23 (17.4)
No. (%) with normal CXR^*a*^	27 (51.9)	38 (47.5)	0.375	8 (42.1)	15 (48.4)	18 (45.0)	22 (51.2)	65 (49.2)
No. (%) with abnormal^*c*^ CXR^*a*^	18 (34.6)	28 (35.0)	0.558	10 (52.6)	14 (44.1)	16 (40.0)	14 (32.6)	46 (34.8)

a Compared by Fisher's exact test.

b Compared by Wilcoxon rank sum test.

c Where specified, abnormalities included pulmonary infiltrates and/or consolidation (*n* = 14), pleural effusion(s) (*n* = 14), hilar and/or paratracheal adenopathy (*n* = 9), cavitation (*n* = 1), and miliary TB (*n* = 1).

Forty-two patients had one or more positive microbiological test results (Fig. 2, bottom). MODS, TEMA, and the proportion method detected multidrug-resistant TB (MDR-TB) in two patients and isoniazid monoresistance in one patient. MDR-TB was identified by TEMA and the proportion method in one patient whose isolate grew on LJ culture but not by MODS. The proportion method reported low-level isoniazid resistance in five additional samples; all others were fully drug susceptible according to all of the methods used.

Positive results were communicated after a median interval of 13 days (interquartile range [IQR], 11 to 18 days; *n* = 57) for MODS and 22 days (IQR, 17 to 28 days; *n* = 78) for LJ culture (*P* < 0.001), and negative results were communicated after a median interval of 41 days (IQR, 38 to 41 days; *n* = 186) for MODS and 63 days (IQR, 59 to 64 days; *n* = 163) for LJ culture (*P* < 0.001). The median time to positivity for patients undergoing TB treatment was 9.5 days (IQR, 6.5 to 21 days; *n* = 6) for MODS and 15 days (IQR, 15 to 17 days; *n* = 8) for LJ culture. Auramine microscopy results were communicated after 1 day for both positive (IQR, 1 to 2 days; *n* = 42) and negative (IQR, 1 to 3 days; *n* = 145) samples. As DST was performed in batches, the time to results is not available; however, laboratory data indicate assay times of 40 to 45 days for the proportion method and 7 to 10 days for TEMA.

Contamination was reported for 38 (26.6%; *n* = 143) direct MODS assays and for none performed following sample decontamination (*n* = 144). For LJ culture, 42 (29.4%; *n* = 143) direct and 4 (2.8%; *n* = 144) decontaminated cultures were reported as contaminated. Sensitivity and NPV were higher following decontamination than with direct processing by all of the microbiological methods used (Table 2). This finding persisted when contaminated specimens were excluded from the analysis. There was no difference in the time to positivity between predecontaminated and directly processed specimens.

**TABLE 2:** Comparison of results and test characteristics for specimen processing following decontamination and direct inoculation of specimens without prior decontamination

Parameter	Auramine microscopy		MODS		LJ culture	
	Decontaminated (*n* = 144)	Direct (*n* = 143)	Decontaminated (*n* = 144)	Direct (*n* = 143)	Decontaminated (*n* = 144)	Direct (*n* = 143)
No. (%) positive	22 (15.3)	20 (14.0)	39 (27.1)	18 (12.6)	46 (31.9)	32 (22.4)
No. (%) negative	122 (84.7)	123 (86.0)	101 (70.1)	85 (59.4)	94 (65.3)	69 (48.3)
No. (%) indeterminate	0	0	4 (2.8)	2 (1.4)	0	0
No. (%) contaminated	0	0	0	38 (26.6)	4 (2.8)	42 (29.4)
All specimens						
% Sensitivity	44.9	40.8	79.6	37.5	93.9	66.7
% NPV	77.9	77.2	90.5	76.0	97.0	88.6
Contaminated specimens excluded						
% Sensitivity	44.9	40.8	79.6	54.5	93.9	91.4
% NPV	77.9	77.2	90.5	82.8	96.8	95.7
Overall						
% Sensitivity	44.9		81.6		93.9	
% NPV	77.9		91.3		96.9	

Paraffin blocks from 121 patients were obtained for full histopathological assessment, and a consensus diagnosis was reached for 117 patients (Table 3). The most frequent histological diagnosis was TB (34.2%). AFB were observed in eight specimens on ZN staining; all were culture positive, and six were positive by auramine microscopy. Interobserver agreement was high for the presence of granulomas (κ = 0.83) and caseous necrosis (κ = 0.90) but not AFB (κ = 0.18).

**TABLE 3:** Histopathological findings and histological diagnoses of patients for whom a consensus diagnosis was reached by two or more pathologists

Result	No. (%) of samples with agreement between ≥2 pathologists (*n* = 117)	Kappa value
Histopathological findings		
AFB on ZN staining	2 (1.7)	0.18
Granuloma	42 (35.9)	0.83
Caseous material	34 (29.1)	0.90
Histological diagnoses		
TB^*a*^	40 (34.2)	0.85
Lymphoma	15 (12.8)	0.66
KS^*b*^	3 (2.6)	0.87
Other malignancy	16 (13.7)	0.88
Hyperplasia	31 (26.5)	0.64
Histoplasmosis	1 (0.85)	0.24
Other	11 (9.4)	0.33

a Where the agreement was between only two pathologists, the diagnosis of a third pathologist was reactive changes (*n* = 3), lymphoma (*n* = 1), non-lymph-node tissue (*n* = 2), or other/unspecified (*n* = 2). Both patients with a non-lymph-node tissue diagnosis were culture positive for TB.

b KS, Kaposi's sarcoma.

This study is the first prospective evaluation of MODS culture for solid tissue specimens. MODS accurately detected TB in lymph node tissue specimens. In contrast to data from respiratory specimens (20), MODS was less sensitive than LJ culture (65.4% versus 76.9%) but almost twice as sensitive as auramine microscopy (36.5%). Comparable to other studies of TB lymphadenitis, the sensitivities of all of the microbiological assays used were lower than those reported for sputum specimens (3, 6, 21, 22), possibly because of the light bacterial load in tissue and/or clumping of pathogens (6). Accordingly, the time to results was longer than that reported for sputum specimens (13 versus 7 days for MODS, 26 versus 22 days for LJ culture) (7). With both culture methods, contamination rates on direct testing were high. Specimen predecontamination increased the sensitivity of all diagnostic tests.

MODS provided accurate data on isoniazid and rifampin resistance simultaneously with diagnosis, which facilitates the timely initiation of appropriate regimens and may prevent further development of resistance. The MDR-TB rate was similar to rates previously documented in Lima (7.7% versus 8.6%) (23), although the sample numbers in this study were small; further prospective testing of greater sample numbers is needed to confirm the accuracy of resistance testing. Universal DST is recommended in regions where the primary MDR-TB rates exceed 3% (24), and MODS may be particularly valuable in such settings.

Physicians have overestimated rates of TB, which can lead to overtreatment and delays in obtaining correct diagnoses. Universal access to MODS has the potential to improve the outcomes of extrapulmonary TB similar to those of respiratory disease (25). Performance of MODS and LJ culture in parallel would combine the benefits of rapidity and simultaneous drug resistance data afforded by MODS and the higher sensitivity of LJ culture.

Nineteen patients had discrepant microbiological and histological results. Similar findings have been reported elsewhere (6). Possible explanations include failure of the host to generate a typical histopathological response, visualization of killed bacilli in patients undergoing TB treatment, and recent use of antibiotics with some antimycobacterial effect (26). Different test modalities may be beneficial for these different patient groups. The concordance between the pathologists' findings and diagnoses was high, although agreement with respect to AFB visualization was poor. The pathologists were blind to clinical information, whereas in practice, findings are interpreted within a clinical context and accuracy may be greater than that observed in this study.

In conclusion, although MODS is less sensitive than LJ culture, it is able to accurately diagnose TB from lymph node tissue significantly faster. It can also correctly detect resistance to rifampin and isoniazid simultaneously with diagnosis, enabling prompt initiation of targeted treatment. No single diagnostic test for TB has all of the properties of an ideal test, and multiple methods should be used in the diagnostic workup of patients with lymphadenopathy. MODS may have an important role to play in the diagnosis of TB in resource-limited settings. These data support the expansion of MODS to solid tissue specimens within programmatic guidelines.
